# A psycholinguistic NLP framework for forensic text analysis of deception and emotion

**DOI:** 10.3389/frai.2025.1669542

**Published:** 2025-10-21

**Authors:** Jonathan Adkins, Ali Al Bataineh, Anthos Khanal

**Affiliations:** ^1^Senator Patrick Leahy School of Cybersecurity and Advanced Computing, Norwich University, Northfield, VT, United States; ^2^Artificial Intelligence Center, Norwich University, Northfield, VT, United States; ^3^Department of Electrical and Computer Engineering, Norwich University, Northfield, VT, United States

**Keywords:** artificial intelligence, criminology, digital forensics, natural language processing, sentiment analysis

## Abstract

Psycholinguistics is an interdisciplinary area of research that bridges elements of linguistics with various branches of psychology. One of its goals is to identify and explain the links that exist between our psyche and the language we speak. In this research, we are expanding upon previous research that we did using several different Natural Language Processing (NLP) techniques to identify persons of interest from a scenario that was generated by a large language model (LLM). We used a different approach to this topic, which allowed us to develop a more nuanced method of reverse engineering and breaking down the psycholinguistic features of each suspect. Through the application of n-grams paired with deception, emotion, and subjectivity over time, we were able to identify and measure cues that can be used to better identify persons of interest from a larger pool of candidates. That dataset was smaller and somewhat limited in scope. We successfully identified the guilty parties from the fictional murder case using a combination of Latent Dirichlet Allocation, word vectors, and pairwise correlations. This research was larger in scope, number of potential suspects, and in the diversity of the corpus used. We were able to determine the guilty parties identified in ground truth using our methodology in this case specifically by focusing on entity to topic correlation, deception detection, and emotion analysis.

## 1 Introduction

The mathematician and philosopher Pythagoras posited in the sixth century BCE that the world and the universe operated in accordance with different types of patterns (Mainzer, 1988). According to the Pythagorean worldview, music was governed by math, the planets in the solar system demonstrated cosmic pattern-driven harmonics, and certain behavior patterns promoted inner harmony (Morales, 2023). Even though some of Pythagoras’s ideas veered into mystical sophistry, some ideas based on his notion of a pattern driven reality are still inherent in science and research. To that end, the domain of Psycholinguistics gives us the tools to find patterns in our speech (written or spoken) that suggest what we may be thinking or planning (Altmann, 2006). The fundamental pattern we are researching is the symmetry between psyche (the mind) and lingua (Latin for language) (Yeager and Sommer, 2012).

In this research, we are applying psycholinguistic analysis to digital forensics for the purpose of identifying key investigative entities or suspects using text they have generated (Soukara et al., 2018). This text could be from E-mails, instant messages, or even transcribed interviews. In this case, we will be applying psycholinguistic theory to a fictitious crime using NLP techniques to identify patterns that suggest culpability in a crime (Alshammari, 2024). Let us say unequivocally that we are not calculating guilt. In essence, we are creating a subset of suspects from a larger population based on key variables. This subset of suspects will be referred to as key entities or suspects. In our research, these variables are the following:

1. Deception over time, calculated using a Python library called Empath.
2. Anger, fear, and neutrality levels in speech over time.
3. Correlation to investigative keywords and phrases.
4. Contradictory narratives.

The research in this document is an expansion of previous research we did using NLP techniques to detect persons of interest using a dataset generated by a Large Language Model (LLM) (Adkins et al., 2024). The dataset in our previous research was smaller in scope, as there were only 10 artificially created characters. In the fictional scenario, one of the 10 characters was murdered and two of the remaining characters conspired to kill him. Using Latent Dirichlet Allocation, word embeddings, n-grams, and pairwise correlations we identified both guilty persons (Liu et al., 2015). For this expanded research, we decided to have an LLM generate a larger fictional scenario. For this experimental model, we had a pool of 18 suspects and two conspirators. The original research used a dataset that consisted of a continuous dialog between the nine suspects that lasted a period of 30 days. In this research, instead of one dialog-driven dataset, we had 18 separate fictional police interviews that were all created using an LLM. The datasets will be discussed in full detail in the methodology section of this paper.

This research presented some interesting challenges due to our use of the LLM-generated police interviews. Specifically, after an initial review of our data we came to realize that the LLM had created two inadvertent problems regarding the 18 datasets. For the sake of full clarity, we had asked the LLM to autonomously pick the identities of the two murderers. One suspect was intended to have normal human reactions to stimuli related to police-related interactions post-crime. These would be a predisposition toward behavior that suggests self-preservation (e.g., presence of deception in speech, increased levels of fear, anger, and subjectivity in answers) (Tomas et al., 2022). The instructions given to the LLM for the second murderer included having a personality that was “more comfortable with lying (Kassin, 2013).” Upon initial analysis of our deception data, we discovered that all 18 fictitious suspects were demonstrating similar levels of deception, meaning the variance between them was very small. This caused us to re-evaluate the approach we were using and implement new analysis techniques. When we applied the new analysis techniques, we were able to “solve the case (Zhou et al., 2004).”

The contribution of this research is a framework of NLP-based techniques that integrate emotion (Mohammad and Turney, 2013), subjectivity (Vrij et al., 2021), narration analysis (Kassin, 2008), n-gram correlation (Zhou et al., 2004), and deception over time to act as a human feature reduction algorithm of sorts (Addawood et al., 2019). It will identify the suspects that are most highly correlated to the crime being investigated. In future research we plan to streamline this approach so that it will only require a small amount of input from the analyst to acquire actionable analytical output. For now, most of the analysis will need to be manual. Our approach will bring to the surface psycholinguistic patterns that suggest a forensic temporal predisposition to certain behavior that may help an investigation when placed in the appropriate context (Petersen, 2010).

## 2 Literature review

In this research we are integrating forensic investigative methodologies with psychology and natural language processing (NLP) to analyze deception in text. The existing research literature is replete with studies that are focused on identifying deception through nonverbal cues such as body language. There are also numerous studies that have explored the role of emotion as a potential indicator of deception. Machine learning techniques have been applied to the analysis of text-based deception since the early 2000s. One of the principal benefits of using ML for research in this area is that it functions by extracting and classifying linguistic features that suggest deceptive behavior. These extracted features can be reused in a variety of different research applications. Based on a comprehensive literature review, we identified three salient areas that are inherent to this domain of research: (1) investigative approaches, (2) identification of linguistic deception in suspect narratives, and (3) emotion as an indicator of deception. The rest of this section will be organized according to these three themes.

### 2.1 Investigative approaches

The studies that fall within this category all have one unifying thread. They all endeavor to formally define scalable methods of extracting cognitive and behavioral features indicative of deception from text. Lebernegg et al. (2024) suggested that fake news could be detected using content analysis and stylistic feature identification. According to their model, consistent use of exaggeration, hedging suggested departure from fact-based language, indicating a higher probability of untruthfulness. Polu (2024) also studied methods of fake news detection. In this model, transformer models such as BERT and RoBERTa were used for contextual credibility. Hancock et al. (2007) studied the use of linguistic features such as pronoun use, negations, and excessive use of sensory descriptions. In this model, deception manifests in subtle but measurable linguistic changes. Since these linguistic changes were very subtle, it was likely not identifiable without the use of computational assistance. Kassin (2008, 2013) studied the use of interrogation techniques and their effectiveness in identifying deception. This technique involved the more traditional police interview, and it was more dependent upon human interaction and analysis of responses from interviewees. The specific tools used in this methodology were verbal coercion and simulated false accusations. Among some of the key findings were that coercive methods were unreliable in identifying facts or determining deception.

### 2.2 Identification of linguistic deception in suspect narratives

The studies that correlate with this category all converge around the following theme: linguistic content, subjectivity, and style exert influence over perception, persuasion and deception. Brzic et al. (2023) identified deception through a machine learning approach that was grounded in psycholinguistic analysis and NLP. The studies used datasets that dealt with the topics of COVID-19 and climate change. The combined features used in the study were N-grams and statistical features captured by the LIWC application. The study used an ensemble of four machine learning classifiers: Logistic Regression, Na¨ıve Bayes, Support Vector Machine, and Random Forest. One of the principal findings of the study was that deceptive language was detectable when using ML. This was especially true when combining psychological and lexical features. Fast et al. (2016) did not explicitly discuss the detection of deception but instead demonstrated how their library Empath could be used to identify deception in text. The Empath library accomplishes this task through statistical comparison with word embeddings and built-in categories. In this particular use case, words that are contextually related to deception are identified in the target text. The words from the target text are then counted and normalized. These tokenized and normalized words are then used as input to train machine learning algorithms. In essence, the Empath library interprets deception through linguistic and lexical cues.

Huang and Liu (2022) indirectly detected deception in their study by focusing on the use of subjectivity versus objectivity in communication. In their study, Huang observed that advertisements characterized by a significant level of subjectivity were often perceived as more trustworthy, even when advertisement claims lacked fact-based information. In this case, “trust” and “subjectivity” served as proxies for deception. Markowitz (2024) also studied the relationship between subjectivity and perception. Markowitz found that there was a relationship between overconfidence and dishonesty. The study found that subjects who were overconfident tended to tell more lies and engaged in cheating behavior.

### 2.3 Emotion as an indicator of deception

Prome et al. (2024) performed a comprehensive review of machine learning based approaches deception detection that use emotion-based cues. The study found that when emotion-based features (when used in context with body language, audio cues, and NLP) were robust indicators of deception. Ren and Ji (2019) studied fake reviews to find emotion-based cues that suggest deception. In their studies they found that emotion cues, especially when used together with cognitive features, improved fraud detection models in domains such as social media. Exaggeration and inconsistent sentiment were the most significant features in identifying deception. Rubin and Lukoianova (2015) used an approach that focused on rhetorical structure coherence to identify deception without directly using emotional features. In their approach, Rubin & Lukoianova incorporated emotional features indirectly through rhetorical relations, especially those associated with evaluation, emphasis, justification, and subjective. Sarzynska-Wawer et al. (2023) used sentiment analysis and statistical negation counts to include emotion into a deception detection technique. Their findings included the following: a shift in emotional tone (increased polarity in lies) suggests that people who lie might inflate statements to sound more appealing or persuasive or appealing.

After a comprehensive review of the literature, including the studies in the three previously discussed areas, we identified several gaps in the research literature. Many of the studies in these three areas cover similar territory. Since 2010 there has been a trend to use machine learning models as part of the overall approach to identifying deception in a forensic narrative. We have identified two fundamental gaps that we will address in this research. The first is that there is a lack of approaches that incorporate multi-layered models that combine emotion, deception, and forensic investigative dynamics. Our approach will use a wide range of tools that complement each other and allow for scalable forensic investigative scrutiny. The second gap that we identified was the lack of a comprehensive model that incorporates an emotion- deception interaction. Through our review of the literature, we have observed that cues in deception are often accompanied by cues in emotion and subjectivity. We will articulate these cues in a temporal fashion so that they will be identifiable on a timeline.

## 3 Materials and methods

In previous research, we explored the use of Natural Language Processing techniques in identifying persons of interest from an LLM-generated scenario that included minimal exposition and dialog between nine suspects over the period of a month. The previous scenario incorporated a limited amount of dialog and exposition. By analyzing emotion scores of each suspect over the one-month period, we successfully identified anomalous spikes in fear, anger, and anxiety. The success of our previous approach was attributed in part to the relatively constrained scope of the scenario that was generated by the LLM. In this study, our goal was to broaden the scale and complexity of the investigation. To specifically address the expansion requirements, we doubled the number of suspects to 18 from the original nine. The textual data we used as our source in this research was also drawn from 18 distinct interviews. These enhancement and expansion changes necessitated some refinement with regard to our analytical techniques. More scalability and nuance were required to effectively approach and analyze this new scenario. In this section we will outline the components of our framework and how they will be used to highlight chronodeceptive and emotosubjective episodes in the transcribed time-series data.

### 3.1 Scenario generation using LLM

The scenario that we used consisted of a set of characters and a peripheral narrative, both of which were fictitious elements that were created according to parameters that we gave to an LLM. The LLM that we used was Google Gemini Flash 2.0 configured with two different system prompts. Two prompts were incorporated into this process to simulate the adversarial nature of an interview between an investigator and a suspect. Conditions were included in the prompt instructions to generate narrative descriptions based on character specific perspectives.

#### 3.1.1 Character creation

A set of 19 inter-related characters were created, all of whom live in the same city. Each fictional character was assigned basic attributes such as age, gender, race, occupation, and personality traits. All these character traits and attributes were assigned at the discretion of the LLM to support the narrative of the murder mystery.

#### 3.1.2 Role assignment

From the pool of characters, two were randomly selected as murders and one as the victim. The random selection process was intended to introduce unpredictability into the narrative and to discourage the reliance on stereotypical tropes commonly found in murder mysteries.

#### 3.1.3 Suspect data enhancements

To build on the overall narrative and individual character attributes, a detailed corpus was compiled for each suspect. This means that for each suspect, a pseudobiographical background was generated. Listed below are some of the pseudobiographical data points that were included for each suspect:

1. Name, age, race, gender.
2. Descriptions of occupation and personality.
3. Personal narratives and internal thoughts related to the murder.
4. Suspected motives and alibis.

#### 3.1.4 Dataset structure

Each police interview was generated and saved as a csv file that was named after the suspect. As the research continued, all 18 of the csv files were aggregated into a single csv file for comprehensive analysis. Each dataset included the following columns:

- Timestamp - These entries were in hh:mm:ss format, starting at 00:00:00. Timestamps were estimated based on typical human speaking speeds, making the timing of the dialog realistic. Interview times lasted from approximately 30 min to 50 min.
- Speaker - Labeled either as the detective or the name of the suspect, as each conversation involved only two participants.
- Text - The full utterance spoken by the identified speaker. Suspect responses were generally longer, reflecting prompt instructions to provide detailed answers, whereas the detective’s dialog was kept short and focused.

### 3.2 Comprehensive detection score

The first technique we planned to implement in this research was the creation of a comprehensive list of all 18 suspects sorted by their calculated deception score. The goal of this technique was to identify any standout suspects who were demonstrating anomalous or unusually high levels of deception. This is not a concrete way of identifying the guilty parties. The intention was to create a short list of five suspects who were standouts regarding the deception they had demonstrated. We incorporated three different pipelines to extract features from the aggregated interviews dataset. Then we used an ensemble of machine learning algorithms to predict the deception scores. The three pipelines we implemented for this research are listed below:

- Empath lexicon for features + ML ensemble.
- SpaCy NLP library & textblob for features + ML ensemble.
- Hugging Face transformer for features + ML ensemble.

#### 3.2.1 Empath lexicon for features and ML ensemble for prediction

In this approach we calculated the comprehensive deception score by using the Empath library in Python to extract features from the text dataset for each suspect. Empath is an NLP tool which uses a lexicon-based approach. Empath has over 200 categories built into its library. Each category has a set of associated words that are based on word embeddings. Table 1 shows an example of some of the categories that are part of the Empath library. When Empath processes a text corpus, it counts the number of words that fall within each of its built-in categories. When the library’s *normalize* parameter is set to **True** a relative word count is provided in a numerical format for each feature. The data that was extracted using Empath is then used to train an ensemble of machine learning algorithms. For this approach we used the following algorithms: XGBoost, Random Forest, Support Vector Machine, Linear Regression and K Nearest Neighbor. The predictions made by each of the algorithms were all averaged together to calculate the overall deception score.

**Table 1:** Feature extraction for empath pipeline.

Empath category	Behaviors/concepts
Emotion and psychology	Anger, sadness, joy, fear, confusion, dispute, envy, nervousness, affection, cheerfulness, pain, contentment, shame
Morality, crime, and law	Violence, crime, law, police, justice, prison, punishment, terrorism, government
Behavior and personality	Emotional, heroic, masculine, feminine, weakness, self, power, dominant, deception, trust
Communication and language	Communication, speaking, hearing, listening, shouting, questions, writing
Social and relationships	Friends, children, family, love, relationships, parties, wedding, divorce
Work and career	Business, office, work, jobs, money, technology, banking, real estate
Health and body	Health, illness, medical emergency, exercise, sleep, body, sexual, injury, fatigue
Time and space	Morning, evening, day, vacation, traveling, urban, rural, home, school
Objects and tools	Weapon, vehicle, furniture, clothing, toys, tools, electronics, machinery
Nature and environment	Animals, plants, weather, earthquake, natural disaster, water, fire, sound
Food and consumption	Eating, drinking, alcohol, cooking, dining

#### 3.2.2 SpaCy and Textblob libraries for features and ML ensemble for prediction

SpaCy is a Python library that functions as a rule-based pipeline for extracting a variety of linguistic features. Textblob is a library which analyzes text primarily for polarity (positive/negative) and subjectivity. Table 2 shows a number of common features that are analyzed in text to determine if a speaker is using deception. SpaCy is a powerful tool used in research and industry to perform a number of NLP tasks. In our second approach to extracting features, we used spaCy to extract first person pronouns (I, my), negations (not, never), modals (may, might, could), named entities (to identify inconsistencies), and average sentence length (to check for complexity). We used the textblob library to identify the levels of subjectivity in the text. The features that were extracted by both textblob and spaCy were combined and normalized so they could be used to train a second ensemble of machine learning algorithms. For the second ensemble we used the following algorithms: Random Forest, Support Vector Machine, XGBoost, K Nearest Neighbor, and Linear Regression. The predictions from each of these algorithms were averaged together. The predictions were used to calculate the comprehensive deception score for each of the 18 suspects. Like the first approach, the results were placed in a table, and they were sorted in descending order by deception score.

**Table 2:** Features extracted by spaCy to detect deception in text.

Feature type	Description	How it is used to indicate deception
Named entities	Detects proper nouns (people, places)	Inconsistent use of locations and names may suggest lying
POS tags	Part of speech tags (verbs, pronouns)	Overuse of such words as could, should, and passive voice may suggest distancing
Dependency parse	Grammatical structure and clause complexity	People who lie may depend on the use of simpler or overly complex structures
Pronoun use	Extraction of first person (I, my) versus third person	Deceptive language frequently includes fewer self references
Negations	Words like not, never	Frequent use may suggest deflection or denial
Adjectives	Descriptive modifiers	Exaggerated and emotional modifiers may indicate falsehoods

#### 3.2.3 Hugging face transformer for features and ML ensemble for prediction

**Figure 1:**
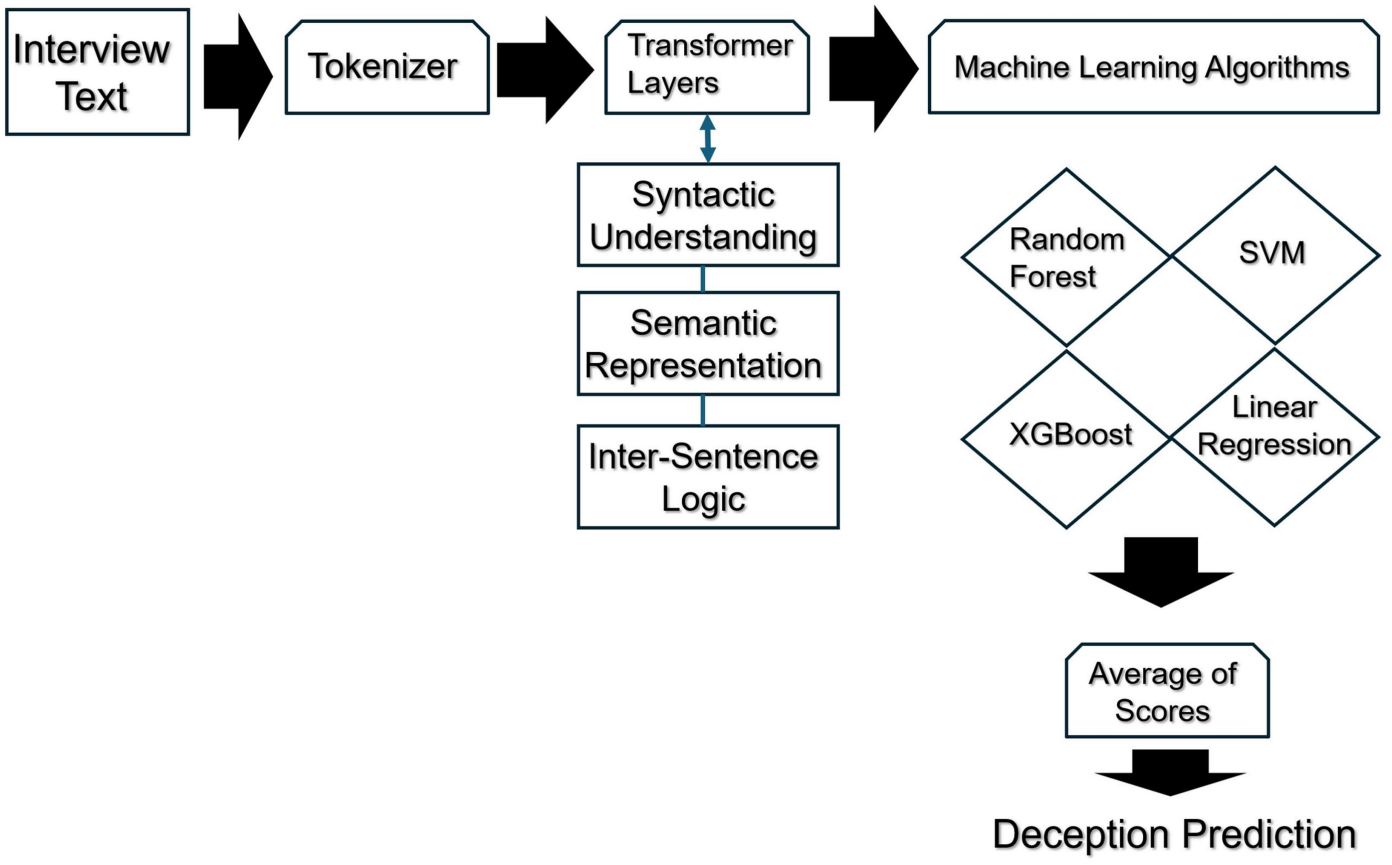
Predicting deception scores using transformer to extract features.

We used the roberta-large-mnli transformer form the Hugging Face community repository to extract features for this third method. If you refer to Figure 1 below you will see the following flow. Interview text from the 18 suspects is used as input to a pre-trained neural network transformer from the Hugging Face repository. The text is preprocessed by being run through a tokenizer, which decomposes each sentence from the suspect’s narrative and isolates each word, treating each word as a separate entity. The tokenized text is then run through several transformer layers for linguistic processing. Features are extracted from this text data in the form of syntactic, semantic, and logical representations which is then normalized so it can be used as input for a machine learning ensemble. The average score from all of the machine learning models was used to calculate the deception scores.

### 3.3 Narrative contradictions

**Figure 2:**
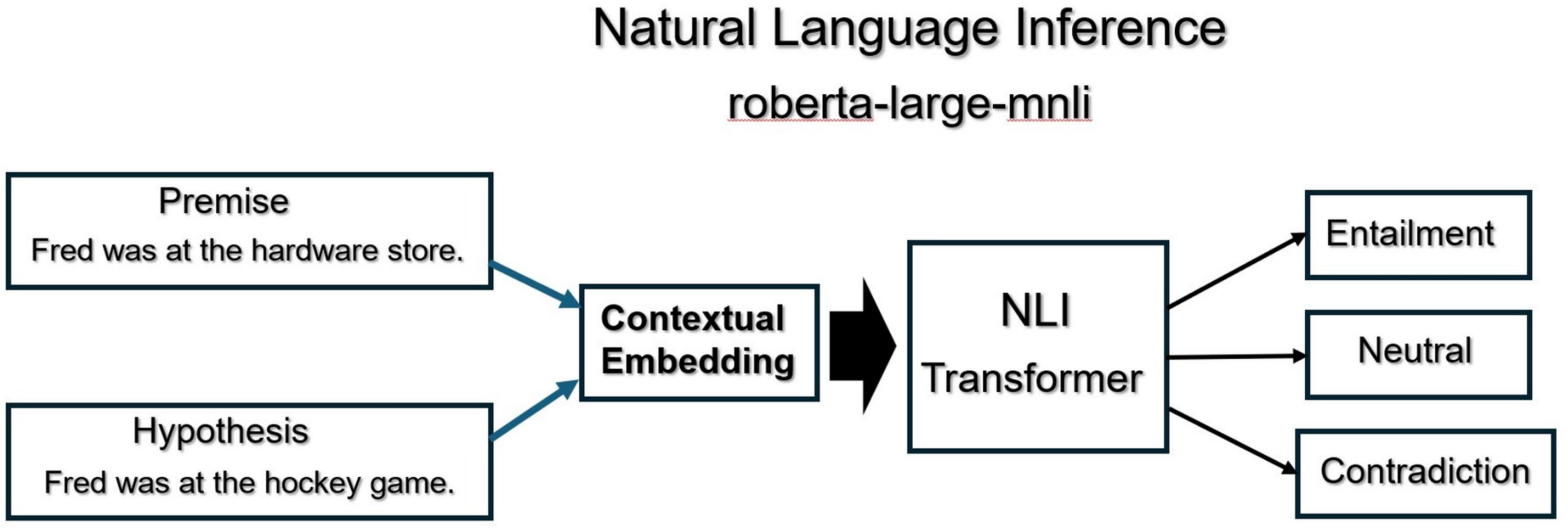
Natural language inference to determine contradictions.

The second technique we implemented as part of the proposed forensic framework was the analysis of narrative contradictions. In the research literature it has been published that people are bad witnesses because they easily led to certain conclusions that may or may not be true. Their recollections of an event may be vulnerable to suggestions (Loftus and Palmer, 1974). One technique for reducing the flaws that exist in witness accounts is to compare several witness accounts side by side. According to many investigators, some attributes of a collective narrative may differ, however the fundamental or key elements of a narrative should be consistent with each other. We used the *roberta-large-mnli* pre-trained transformer from Hugging Face to analyze all the statements from the individual suspects for discrepancies and contradictions. The process is detailed visually below in Figure 2. First, statements that include named entities and times are extracted from each of the suspect’s answers. Statements are embedded using the transformer. Statements which involve similar named entities and times are compared using Natural Language Inference. Specifically, one statement is encoded as a *Premise* and the second as a *Hypothesis*. During the comparison, if the two statements contain contradictory language, the Premise/Hypothesis comparison is labeled as a *Contradiction*. If the comparison of the two statements is True, then the comparison is labeled as an *Entailment*. If the comparison is neither true nor false, then the label is Neutral.

### 3.4 Keyword to entity correlation: suspicion

**Figure 3:**
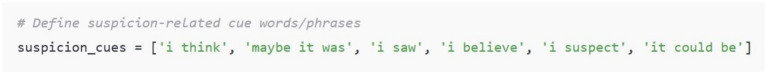
Python list containing suspicion-related phrases.

**Figure 4:**
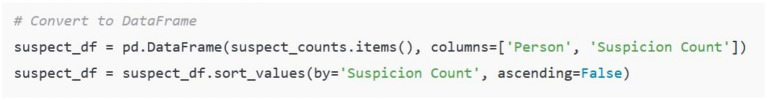
Python dataframe containing the suspicion person to phrase mappings.

In our previous research, we used person to keyword correlation in several different implementations to identify those suspects who had a stronger correlation to investigative keywords than others (Adkins et al., 2024). In the dataset we are currently using, we saw that in each of the suspect interviews, the detective asked the interviewee whom they perceived as being “suspicious.” Based on this observation, we decided to implement entity to keyword correlation for the current use case. If you look at Figures 3, 4 below you will see how we achieved this implementation. As shown in Figure 3 we used Python to create a list of suspicion-related linguistic cues. This included phrases such as “I think,” “I suspect,” and “maybe it was.”

As seen below in Figure 4, each individual suspect from the dataset was mapped to a concrete number of phrases that were identified when a person’s name was mentioned in the same context as the target phrase. All of the mappings were then stored into a dataframe called suspect df. The contents of this dataframe were then displayed as a bar graph sorted in descending order.

### 3.5 Deception timelines

**Figure 5:**
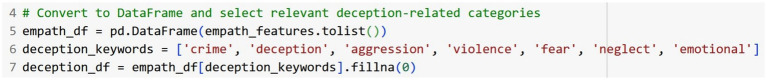
Deception related features used by Empath.

**Figure 6:**
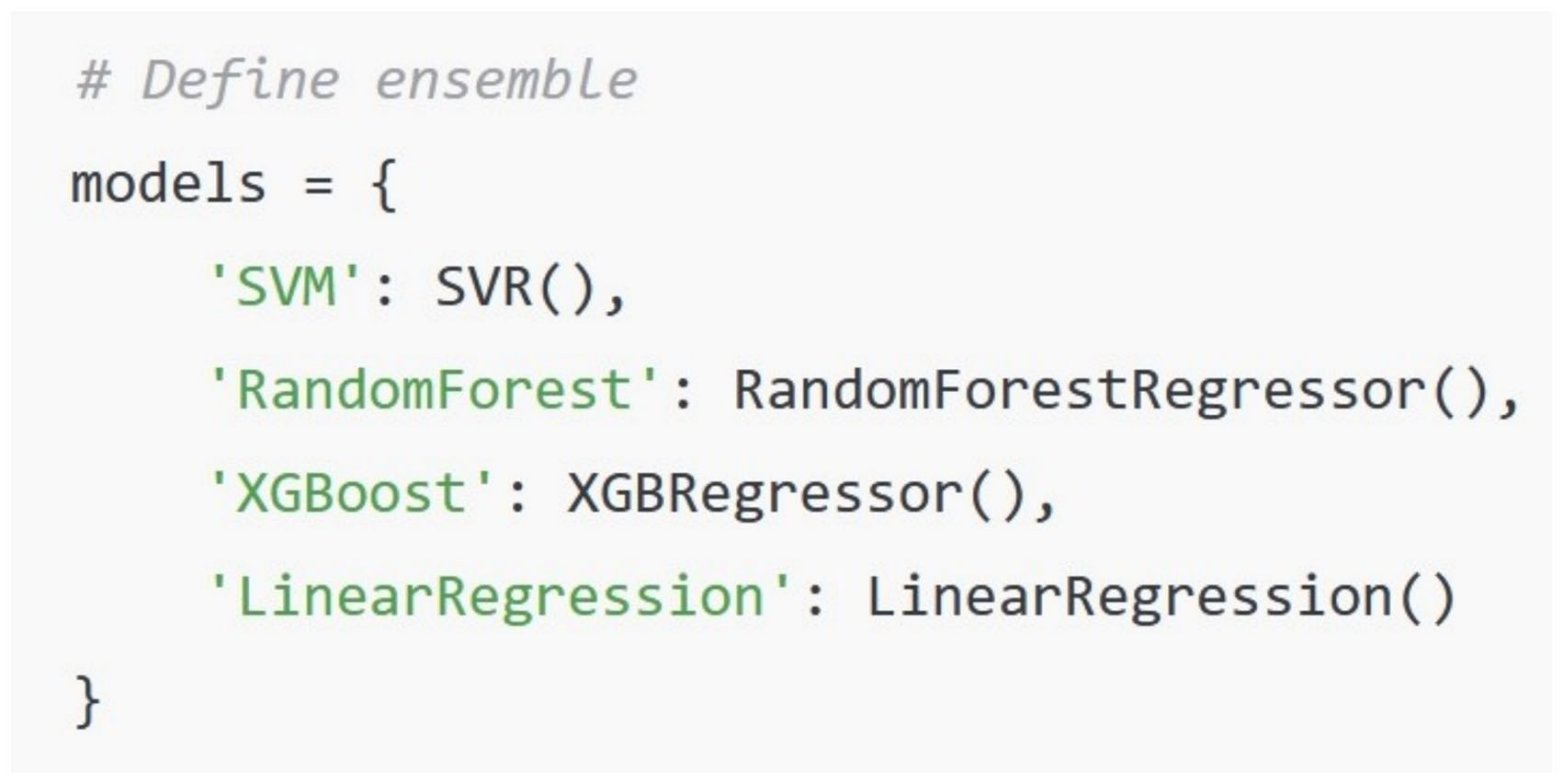
Machine learning ensemble for predicting deception scores.

Our methodology in practice adopts a progressive refinement strategy for psycholinguistic investigation. We begin by identifying general patterns and then we progressively move toward specific targets. After we identify an initial subset of suspects who exhibit strong correlations to investigative keywords, we shift to a detailed examination of individual timelines using the analysis of emotional expression, subjectivity, and linguistic deception. The first timeline we considered was the deception timeline. We calculated deception using one of the previous methods we used for calculating the overall deception score. We used the Empath library in Python to extract features from the text for a suspect’s interview. As shown in Figure 5 below, we provided several keywords that fell within the same semantic vector space as “deception.” The Empath library then normalized the words in the target text that it found. The normalized features were then used as a training dataset for an ensemble of machine learning algorithms (Figure 6) which predicted the deception score for each timestamp. The result was a continuous line trajectory displayed on a graph with all the timestamps shown on the x-axis. If any of the suspect replies contained psycholinguistic patterns consistent with deception, this would be suggested in the graph by a peak that was higher than others in the graph.

### 3.6 Composite emotion time series graph

In our methodology, identification of deception in speech only tells part of a story. We know that someone is attempting to be untruthful or perhaps manipulative, however, more layers of information can fill in the blanks and provide more validation that deception is in fact taking place. For example, if a suspect demonstrates a high manifestation of deception when asked about an alibi (for example, “I was never at the coffee shop on June 12th”), this looks a little bit suspicious. However, if at that same moment, the suspect demonstrates a large peak in subjectivity and in anger when all other replies were baseline, then it suggests the following. The individual is likely attempting to legitimize deception by introducing non-factual, opinion-based and potentially misleading information. Research literature in the domain of investigative techniques also suggests that the sporadic bursts of anger often accompany deception (Newman et al., 2003). To provide some additional layers of depth to our individual timeline analysis, we created a composite graph that displays levels of anger, fear, neutrality, and subjectivity across time. The timestamps are shown on the x-axis, and a horizontal trajectory line is shown for each of these variables in their own isolated graphs that stacked vertically. The scores for anger, fear, neutrality, and subjectivity were all calculated using Hugging Face pre-trained transformers.

## 4 Results

To investigate the psycholinguistic patterns within the data, we employed a multi-tiered NLP-based analytical framework designed to isolate features of interest within the interview narratives. We started with an initial approach that that involved creating a short list of the top five suspects from the original pool of 18 based on elevated levels of linguistic deception. According to our original model, we would then further scrutinize this top five suspect list by conducting detailed analysis of the individual narratives focusing on indicators such as deception over time, anger, fear, and contradictions in provided details. After generating three lists, we encountered an issue during the suspect selection process. This issue involved the notion that the artificially generated police interviews lacked essential anthropic features common to average people. Specifically, the artificial character responses in the interviews lacked features that are intrinsic to human cognition and behavior. These features include behavior such as memory-related errors that are quintessentially manifest as discrepancies or contradictions in suspect alibis. We will discuss these limitations in greater detail in the Challenges subsection of this document.

### 4.1 Challenges

This research relied on an artificially generated murder investigation scenario based on parameters that we provided to an LLM. Some of these parameters included the names and genders of 18 fictional people. The parameters also included the identities and basic psychological profiles of the two guilty conspirators. According to our instructions provided to the LLM, one of the murderers was to have an average response to stimuli related to anxiety and fear. The second conspirator was to have a slightly more morally pliable profile where they were more comfortable with the use of deception and lying. Several investigations often depend on the existence of details from a narrative that are out of place or contradictory in order to unmask a suspect who is being intellectually elusive or deceptive. There were no direct or obvious contradictions or discrepancies in the narratives provided by the LLM. Originally, we kept the identifies of the two conspirators unrevealed until we created our initial short lists. When we reviewed the ground truth, our results were mixed at best. Out of three different methods, one list showed both guilty conspirators in the top five. The other two showed them within the top ten but not in the top five. Upon further scrutiny of the numbers, we saw that all 18 suspects had relatively high deception scores and a low variance between them. This suggested to us that everyone was being untruthful to some degree. We decided to take a different approach and under conditions where all the suspects were uncooperative, use reverse engineering to identify what it was that made the suspects guilty. The answer we found further supported the idea that the dataset was lacking in anthropic features. Furthermore, the remaining tools we used proved to be effective in conditions where certain variables were uncooperative or absent.

### 4.2 Comprehensive deception list

We used three different methods of extracting deception features and then used an ensemble of machine learning algorithms to predict the order of the 18 suspects. Our first method used a Python library called Empath to extract features from the text that would be used to train five algorithms. Empath is a lexicon-based NLP library that has several pre-defined categories that are based on word embeddings. The pre-defined categories include violence, negativity, crime, affection, work, money, and emotions. When Empath analyzes a sample of text, it returns a score based on how many words in the sample appeared under each category. Since Empath is lexicon-based it suffers from the limitation that it can’t understand context of words. However, when it is used along with machine learning algorithms, its efficacy improves. We aggregated all 18 of the police interviews, then used Empath to extract features. We then used the following algorithms as an ensemble: Random Forest, Support Vector Machine, KNN, XGBoost, and Linear Regression. The outputs of the ensemble were averaged. The overall deception scores were predicted for each suspect. The table was sorted in descending order based on their deception score. Table 3 below shows the output of the Empath method we used for creating a sorted list based on overall level of deception.

**Table 3:** Overall deception score using empath and machine learning ensemble.

Rank	Speaker	Deception score (%)
1	Tasha Freeman	0.72%
2	Samuel Linwood	0.71%
3	Owen Bishop	0.70%
4	Gabriela Rios	0.69%
5	Elliot Moore	0.67%
6	Janice Wu	0.65%
7	Avery Johnson	0.64%
8	Sophie Kramer	0.63%
9	Nina Patel	0.62%
10	Priya Shah	0.59%
11	Darren Shepard	0.56%
12	Clara Bennett	0.56%
13	Daniel Yazzie	0.54%
14	Jordan Ellis	0.54%
15	Marcus Delgado	0.54%
16	Veronica Cortez	0.54%
17	Luis Moreno	0.53%
18	Trent Holloway	0.51%

According to the scenario generated by the LLM, Owen Bishop and Gabriela Rios were the identities of the two conspiring murderers. When using the Empath method of creating the short list, these two suspects came in third and fourth place regarding level of deception in their speech. If you look at the scores for all the suspects in the list, you will see that all of the suspects were within the 50 threshold to be considered *deceptive* in their speech. According to this model’s output, there is only a 20% difference between the top and bottom members of the list.

The second model we implemented to score the pool of suspects for deception used spaCy and textblob libraries in Python to extract features. The spaCy library is an industrial pipeline option for NLP operations that can perform tasks such as named entity recognition, dependency parsing, and part of speech tagging. Textblob is a more basic library which can perform polarity analysis (positive/negative) and subjectivity analysis. Several features were extracted using each of these two NLP solutions. As with the previous model, Support Vector Machine, KNN, Linear Regression, XGBoost, and Random Forest were used to predict the deception scores. The averages of the collective outputs were used to make these computations. Table 4 shows the second method we used to determine overall level of deception sorted in descending order.

**Table 4:** Overall deception score using spaCy, Textbloob, and machine learning ensemble.

Rank	Speaker	Deception score
1	Janice Wu	0.513
2	Sophie Kramer	0.511
3	Jordan Ellis	0.504
4	Elliot Moore	0.503
5	Daniel Yazzie	0.500
6	Gabriela Rios	0.497
7	Priya Shah	0.497
8	Clara Bennett	0.496
9	Luis Moreno	0.496
10	Owen Bishop	0.493
11	Samuel Linwood	0.492
12	Darren Shepard	0.491
13	Trent Holloway	0.486
14	Avery Johnson	0.486
15	Tasha Freeman	0.484
16	Nina Patel	0.474
17	Veronica Cortez	0.462
18	Marcus Delgado	0.460

In Table 4 our two guilty conspirators have different rankings. Gabriela Rios is ranked at number six and Owen Bishop is at number 10. However, if you look at the deception scores for this table, the small variance is much more noticeable. Using the spaCy and textblob features, the average prediction scores ranged only between 46 and 51%. The average score in this table is lower than the table for the Empath library. The lower scores and different rankings for Owen Bishop and Gabriela Rios demonstrate that there is a difference in the way that the two models approach scoring sentiment and other features that determine deception.

We created one more model to score the pool of suspects for overall deception. In this last model we used a pre-trained transformer from Hugging Face to extract features for deception. When transformers extract features for deception, they do not use lexicon-based features like “anger” or negations. Instead, they will extract vector embeddings that will capture cues, emotional subtexts, and semantic patterns. For this last table we used the *roberta-large-mnli* transformer for feature extraction. The same five machine learning algorithms were used to predict the scores for the 18 suspects. The sorted list for this model can be seen in Table 5.

**Table 5:** Overall deception score using hugging face transformer and machine learning ensemble.

Rank	Speaker	Deception score
1	Nina Patel	0.922
2	Samuel Linwood	0.922
3	Marcus Delgado	0.918
4	Priya Shah	0.918
5	Sophie Kramer	0.911
6	Jordan Ellis	0.910
7	Owen Bishop	0.901
8	Tasha Freeman	0.899
9	Gabriela Rios	0.898
10	Clara Bennett	0.881
11	Trent Holloway	0.880
12	Daniel Yazzie	0.879
13	Veronica Cortez	0.876
14	Luis Moreno	0.854
15	Janice Wu	0.850
16	Avery Johnson	0.812
17	Elliot Moore	0.803
18	Darren Shepard	0.796

In this third model, Owen Bishop appears in the number seven ranking, and Gabriela Rios appears in the number nine rank. As in the previous model, both conspirators appear in the top 10, however their placement is different. Also, the variance between the other suspects is even tighter than the other two models. Fundamentally, the range of scores is between 8 and 9. Other than the fact that Gabriela Rios and Owen Bishop appear in the top 10 in all three of these models, there is no consistency. There is also no standout suspect regarding the amount of deception demonstrated. Depending on the tool that is used, you could see a slightly different outcome in every instance and new model. For this reason, we decided to change our approach to identifying key suspects using other tools from our proposed framework.

### 4.3 Narrative contradictions

After we determined that the deception score approach was not a viable method, we decided to focus on contradictions in suspect narratives. For this approach, we used a Natural Language Inference (NLI) transformer from Hugging Face. The specific model that we used was roberta-large-mnli. In this method we used Python to extract key entities and dates from suspect interviews. Those statements were clustered and organized by date. Similar statements would be compared and if there was a contradiction between the two statements, it would be identified by the algorithm. An example of the previously discussed method would be as follows. A statement extracted from Owen Bishop’s interview might be: “I was at the coffee shop on Monday, June 12, at 3 p.m.” A statement from Gabriela’s interview might say: “Owen was at his favorite sports bar on Monday, June 12, at 3 p.m.” Both hypothetical statements would likely reside in the same cluster since they articulate similar ideas using the same date and time. Since there is a discrepancy between Owen’s and Gabriela’s statements regarding the location, this would be identified as a “contradiction.” Since we know from ground truth that Owen Bishop and Gabriela Rios are the murders, we compared the interviews of the two suspects using clustering and NLI. There were no obvious contradictions found in any of their statements. The only “contradiction” that existed between the pair of narratives was that one suspect would make a statement that included a date and time and the other simply omitted any mention of this statement. The systematic lack of any misalignment of peripheral facts suggested the LLM’s sterile representation humans who lacked anthropic or fallible traits. We concluded after these results that a focus on narrative contradictions was also not a viable option. We decided to focus next on whom the 18 suspects each perceived as guilty.

### 4.4 Keyword to entity correlation: suspicion

**Figure 7:**
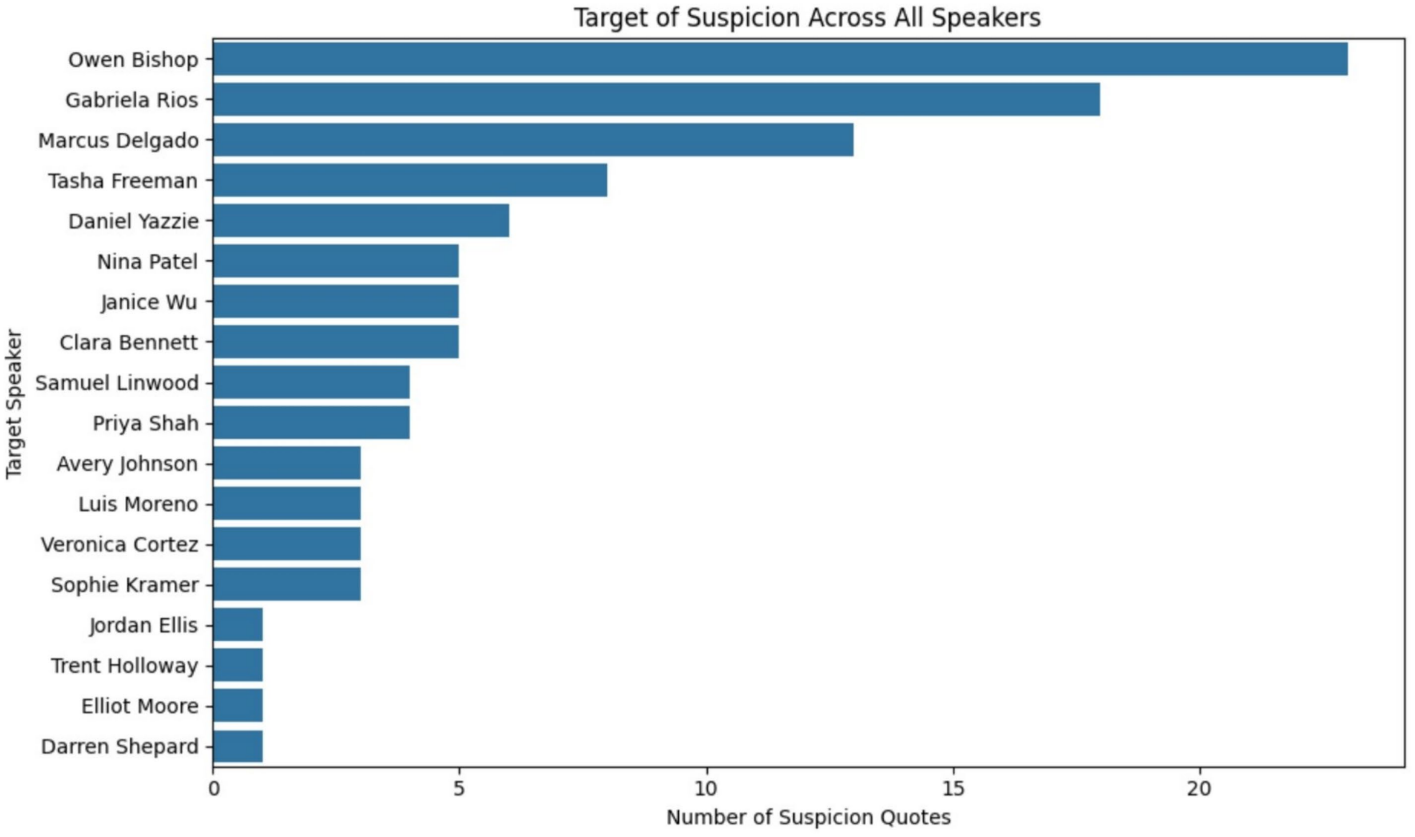
Suspicion keywords mapped to each suspect.

For this technique we used Python to extract suspicion related words from each of the suspect interviews. Each of the suspicion related words was then mapped back to a target individual. This mapping was then included in a dataframe so it could be articulated as a graph. If Janice mentioned that Gabriela was a suspect, then this data point was captured and mapped. If Clara stated that Owen “looked like a suspect,” then this correlation was mapped. A bar graph was then generated to display which of the 18 suspects was the target of the most suspicion by the other individuals who were being interviewed. If you look at Figure 7 you will see that Owen Bishop is the person whom most of the others suspect of being guilty. Gabriela Rios is the second most suspected person. The motive of the LLM in selecting its two conspirators for this case appears to be the fact that everyone else suspected them. This is obviously not actionable evidence that could push an investigation forward, but it does (in this case) solve our fictitious crime. If comprehensive deception does not provide a clear short list of suspects and there are no narrative discrepancies regarding alibis or official data points, the logical next step is to scrutinize individual timelines, which is what we will discuss next.

### 4.5 Deception time series composite with bigrams

This was the first of two techniques we used to analyze a suspect’s timeline juxtaposed with a specific variable. In this case, the variable is deception. For each suspect, their interview was articulated as a graph with their answer timestamps on the x-axis. The answer that was provided at each timestamp was assessed for levels of deception using the Empath library (for feature extraction) and an ensemble of machine learning algorithms to predict the deception score for that timestamp. The deception levels were depicted on the graph as a continuous line. The deception scores are shown on the y-axis of the graph. The continuous line graph is accompanied by a table which shows a list of meaningful bigrams in one column and the timestamp at which they appear in another column. The significance of the bigrams is determined by whether they occur at times when levels of deception are high. The high mark is determined by a threshold value which is set in this case at 0.4, which is approximately 40% level of deception. Anything above this threshold suggests use of language that is out of bounds of routine speech and which suggests a legitimate attempt at deception between speaker and receiver.

**Figure 8:**
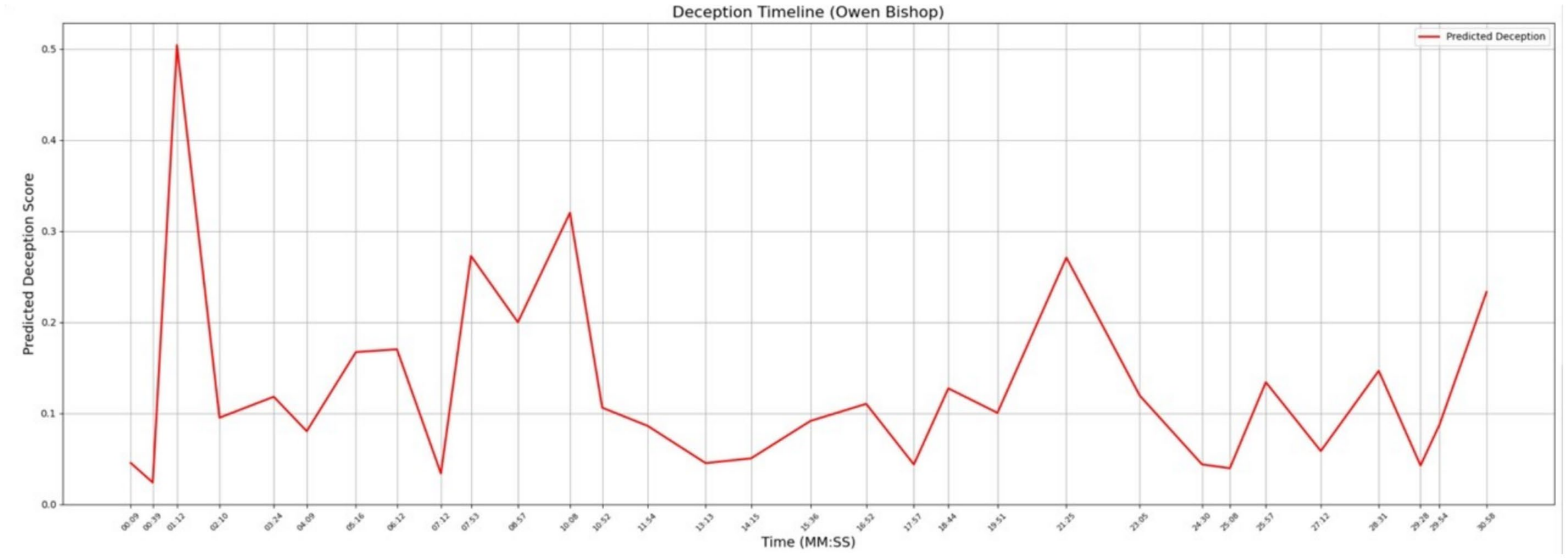
Deception timeline for Owen Bishop during interview.

In Figure 8 you can see the levels of deception for Owen Bishop that correlate to the timestamps on his police interview. Table 6 shows the topics that were discussed at times when the deception levels were peaking. This information needs to be assessed in full context for it to be valuable to an investigator. There is one data point of interest in this graph. There is a significant peak at timestamp 01:12 where the topic of discussion was “Disagreements Leah.” For full context, we reviewed the original transcribed interview for the answer that was provided at this timestamp. According to Owen’s answer, the “disagreements” that were cited at this timestamp had to do with professional differences in a development project that was taking place in the Old Market District. This peak stands out since all the other data points fall within lower tolerances of the deception score.

**Table 6:** Significant bigrams in Owen Bishop’s interview.

Bigram	Timestamp
Maple street	00:09
Street apartment	00:09
Disagreements leah	01:12
Old market	01:12
Market district	01:12
Old market	02:10
Market district	02:10
Late night	02:10
Question detective	04:09
Late night	06:12

**Figure 9:**
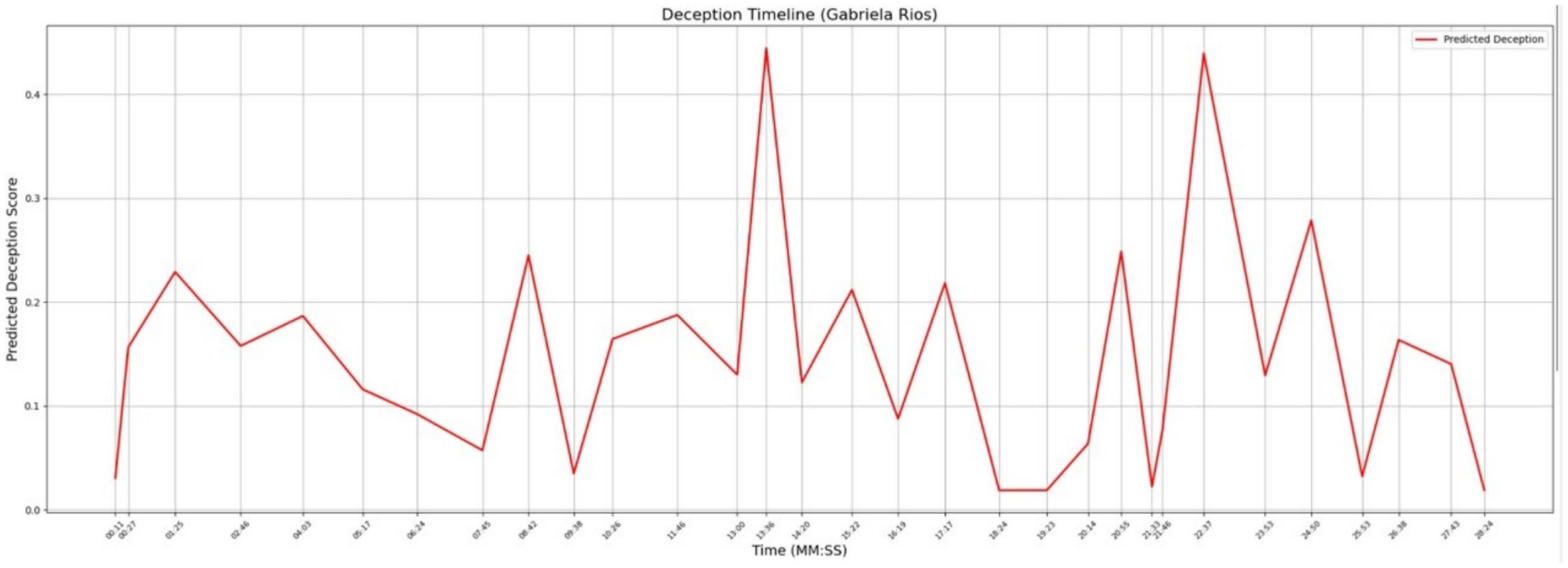
Deception timeline for suspect Gabriela Rios.

Figure 9 and Table 7 show the significant bigrams and deception levels for Gabriela Rios, who is the second murderer according to the LLM-generated scenario. There are two notable examples we will point out in these two visuals. Specifically, there are two points in Gabriela’s timeline where the level of deception reaches the top of the graph. The first takes place at timestamp 13:36, where the topic is “potential consequences.” The second maximum point takes place at 22:37, where the topic is “Officer Whitaker.” In the first elevated peak, Gabriela explains to the detective why she had been arguing with the victim. In the second peak, Gabriela explains how she wrote an unflattering story about Officer Whitaker, saying he used unnecessary force. When both topics are considered in context when juxtaposed to the levels of deception, the data suggests that Gabriela is being untruthful in her statements (Table 8).

**Table 7:** Significant bigrams in Gabriela Rios’ interview.

Bigram	Timestamp
Coffee shop	10:26
Daily grind	10:26
Remember seeing	10:26
Potential consequences	13:36
Leah story	17:17
Daily grind	18:24
Oak street	19:23
Officer Whitaker	22:37
Yes detective	26:38
Verify information	26:38

**Table 8:** Results table for all techniques.

Investigative technique	Results
Comprehensive deception score	Empath, spaCy + textblob, and Hugging Face transformer methods of feature extraction resulted in three separate sorted lists that showed very little variance in deception for all 18 suspects
Narrative contradiction analysis	No contradictions or discrepancies were identified in any of the narratives
Bigram to entity correlation: suspicion	Owen Bishop (#1) and Gabriela Rios (#2) were perceived as the most suspicious people out of the pool of available candidates based on suspicion words mentioned in relation to a specific name
Deception timeline	Owen Bishop showed one significant spike in deception in his interview timeline at timestamp 01:12. Gabriela Rios showed two significant spikes in deception at timestamps at 13:36 and 22:37
Composite emotion and subjectivity graph	Owen Bishop’s graph showed spikes in both subjectivity and in anger at 01:12, right at the timestamp where deception spiked. Gabriela Rios’s graph showed elevations in subjectivity, fear, and anger at 13:36 during her first spike in deception During her second spike in deception at 22:37, she showed a *significant* spike in anger

### 4.6 Composite time series graph for anger, fear, neutrality, and subjectivity

**Figure 10:**
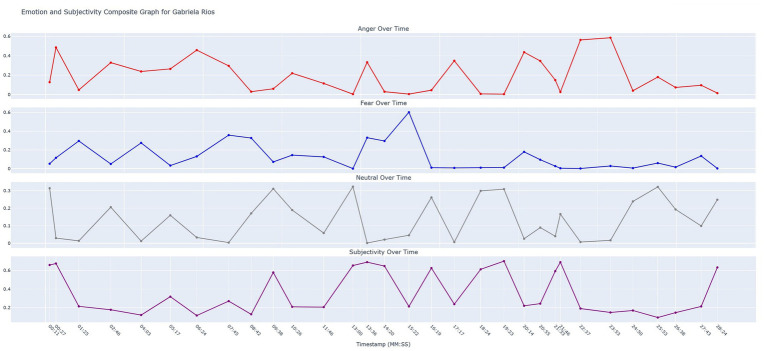
Emotosubjectivity graph for Gabriela Rios.

In addition to the time-series deception graph, we created a composite graph that shows levels of anger, fear, neutrality, and subjectivity over time. Chronodeceptive data (topic bigram data layered with deception scores in a time-series) provides only partial information. It must be considered in context with other variables such as the amount of anger, fear, and subjectivity the suspect is demonstrating. For example, we know that Gabriela is being deceptive when she is answering a question that deals with a “potential consequences.” There is a significant spike in the level of subjectivity and an increase in the level of anger which further supports the suggestion that Gabriela is using deception when answering this question. The research literature has shown that spurious anger sometimes accompanies deception. Furthermore, high levels of subjectivity are often seen as cues for deception as the answers are veering away from fact-based speech and toward speech that is more heavily laden with opinion and fewer facts. We refer to this composite graph as time-series emotosubjectivity. Figure 10 below demonstrates the emotosubjectivity for Gabriela Rios.

Since the identities of the two LLM-generated murderers were determined previously through keyword reference correlation (see Figure 7), we will not elaborate on the emotosubjectivity trajectories for Owen. Instead, we will close out this section by demonstrating some of the salient psycholinguistic features that can be extracted from this composite graph using some key data points taken from Gabriela’s deception timeline. In Figure 9 (Gabriela’s deception timeline) the bigram “potential consequences” appears at timestamp 13:36. The bigram “Officer Whitaker” appears at timestamp 22:37. These bigrams all occurred at points when levels of deception were notably elevated. We observed after cross-referencing the emotosubjectivity graph that at 13:36, Gabriela’s subjectivity crested, suggesting the presence of non-factual or speculative content.

## 5 Discussion

The scenario that we used that was generated by the LLM allowed us to simulate realistic investigative interactions with suspects in a case without the legal and ethical constraints normally associated with real-world sensitive and confidential data. The problem we encountered was that the dynamics involved in the case were lacking genuine human nuances that produced the genuine psychological feedback necessary to fully test our NLP-based framework. The first obstacle we encountered was the fact that our sorted comprehensive deception score table showed that everyone in the pool of candidates was being deceptive to a certain degree. The amount of variance between the 18 suspects was so significantly small that no one person stood out. We had gone on with the assumption that those responsible for the murder would have a higher overall deception score. Even if additional suspects had proven to be more deceptive, there would be a short list of 10 or fewer suspects who could be candidates for closer scrutiny. Our second approach proved to be equally ineffective at identifying any standout suspects, as there were no identifiable contradictions in statements, not even subtle ones. Our third attempt proved more successful, since the murderers listed in ground truth were identified using suspicion keywords. Other than the high level of suspicion directed toward Owen Bishop and Gabriela Rios of the crime (see Figure 7) show, there was no substantiating evidence other than the high level of deception associated with Gabriela’s comments regarding the “potential consequences” and “Officer Whitaker.” We found the emotosubjectivity graph to be a useful tool to cross-reference with other time-series data. We have noted previously that that emotion or deception related data taken individually is not very useful. When all these factors are considered collectively in context, it provides greater insight into human behavior that may not be readily apparent by simple auditory interpretation of a suspect’s response to a question. In addition to our findings, other emerging technologies may offer complementary support for forensic analysis. AI-enhanced blockchain has been proposed as a way to improve the security, transparency, and reliability of digital investigations (Ressi et al., 2024). While not directly related to psycholinguistic analysis, such approaches could strengthen chain-of-custody and reproducibility, and they may align with future extensions of our framework.

## 6 Conclusion and future work

This research introduced a systematic and practical NLP-based framework for identifying candidates from a broader suspect population during a forensic investigation. Our methodology incorporates several variables for consideration. These variables include n-gram correlation, emotion, subjectivity, narrative contradictions, and level of deception. When analyzed in time-series graphs these variables provide a glimpse into a suspect’s state of mind as well as how strongly they correlate to specific investigative topics of interest. This is not assigning guilt but rather implementing psycholinguistic techniques to identify salient actors in a case and help forge a solid narrative timeline. For n-gram analysis, we focused on bigram extraction where the bigrams suggested a target suspect and identified alibis. For emotions, we used pre-trained transformers from Hugging Face to identify anger, fear, and neutrality. We used another transformer to identify levels of subjectivity in text. The anger, fear, neutrality, and subjectivity were mapped over time in a composite graph where timestamps from police interviews were placed on the x-axis. Levels of deception were mapped over time on a separate graph using the Python “Empath” library and an ensemble of machine learning algorithms to predict the scores. Natural Language Inference transformers were used to identify any contradictions in suspect narratives. Relevant bigrams were plotted on a time- series graph along with levels of deception as a second layer. We were able to calculate the identities of the two guilty suspects using target suspect bigrams. In short, according to the LLM, the two people whom others suspected were in fact guilty. While we were successful in solving fictional crime using our methodology, it did not get the rigorous evaluative assessment we had hoped. Our methodology allows us to explore (to some degree) several latent psycholinguistic cues that can lead investigators to concretely identify a primary suspect. We believe that the shortcomings from our LLM-generated murder scenario come from the model’s inability to sufficiently emulate the human-centric process that drives decision making. Specifically, an AI model can’t adequately simulate irrational human thought patterns that arise during the aftermath of a crime. The process of contriving alibis, keeping the emotions of anxiety and fear under control, and the use of deception in furtherance of self-preservation are not features common to deep learning models. In future research, we plan to expand this research with human subjects and EEG data, and also explore AI-enhanced blockchain for forensic frameworks.

## Data Availability

The raw data supporting the conclusions of this article will be made available by the authors, without undue reservation.
